# Supplementary material for: The Combination of Gold and Silver Food Nanoparticles with Gluten Peptides Alters the Autophagic Pathway in Intestinal Crypt-like Cells
Source: Int J Mol Sci. 2023 Aug 22;24(17):13040. doi: 10.3390/ijms241713040 (PMC10487529; doi:10.3390/ijms241713040)
Supplement: Supplementary file 1 [file ijms-24-13040-s001.zip › ijms-2561762-supplementary.pdf]

Supplementary material

The Combination of Gold and Silver Food Nanoparticles with Gluten Peptides Alters the Autophagic Pathway in Intestinal Crypt-like Cells

Clara Mancuso [1,2], Eric Tremblay [2], Elisa Gnodi [1], Steve Jean [3], Jean-François Beaulieu [2] and Donatella Barisani [1,*]

1 School of Medicine and Surgery, University of Milano-Bicocca, 20900 Monza, Italy; clara.mancuso@unimib.it (C.M.); elisa.gnodi@unimib.it (E.G.)

2 Laboratory of Intestinal Physiopathology, Faculty of Medicine and Health Sciences, Université de
Sherbrooke, Sherbrooke, QC J1H 5H4, Canada; eric.tremblay@usherbrooke.ca (E.T.); jean-francois.beaulieu@usherbrooke.ca (J.-F.B.)

3 Department of Immunology and Cell Biology, Faculty of Medicine and Health Sciences, Université de
Sherbrooke, Sherbrooke, QC J1H 5H4, Canada; steve.jean@usherbrooke.ca

* Correspondence: donatella.barisani@unimib.it; +39-0264488304

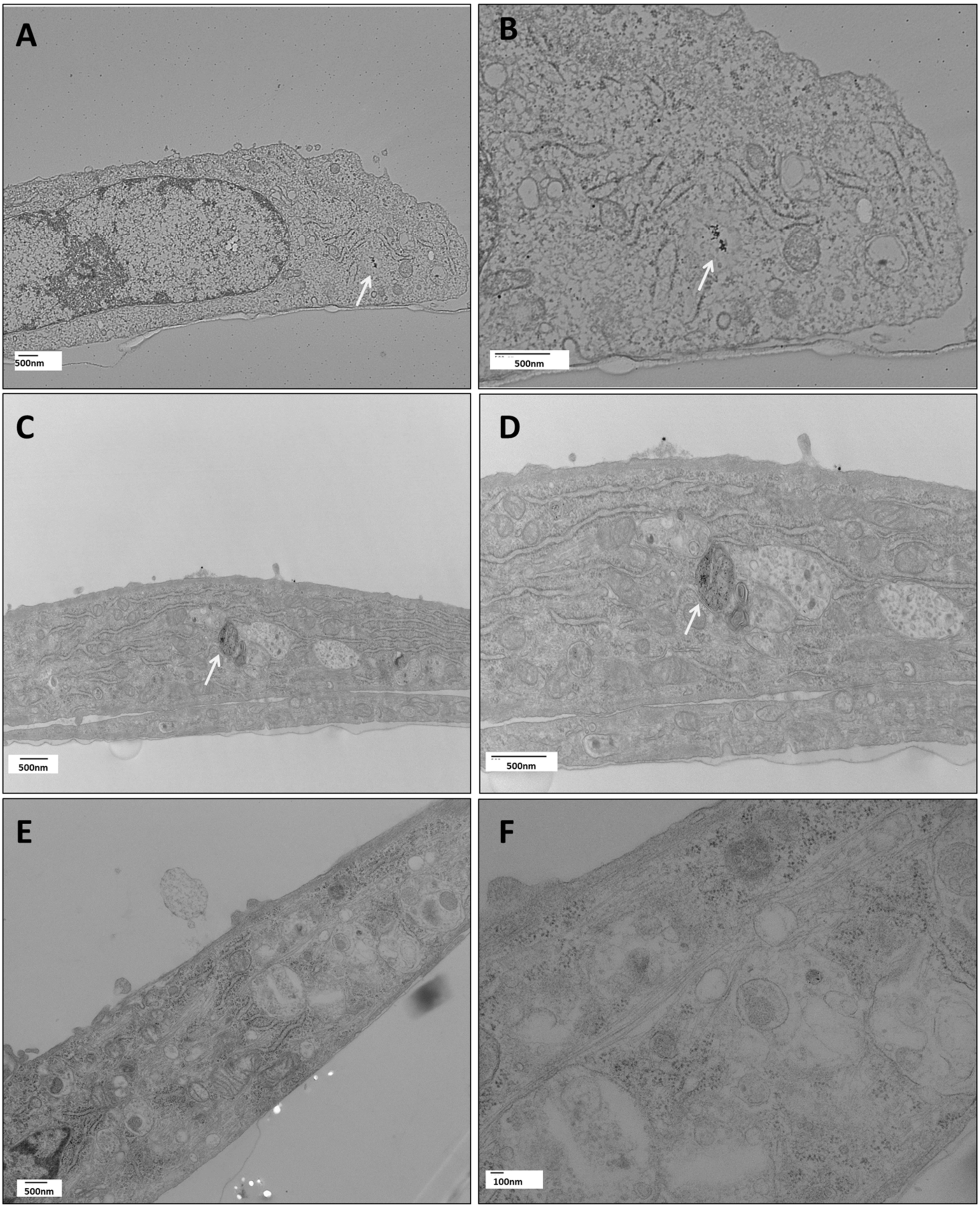

Figure S1. *TEM images of HIEC cells exposed to: A,B) AuNPs 25µg/ml for 24 hours; C,D) AgNPs 5µg/ml for 24 hours; E,F) Bafilomycin 50nM for 2 hours. Scalebars are reported on each panel. Images were obtained with an HITACHI H-7500 TEM after proper fixation.*

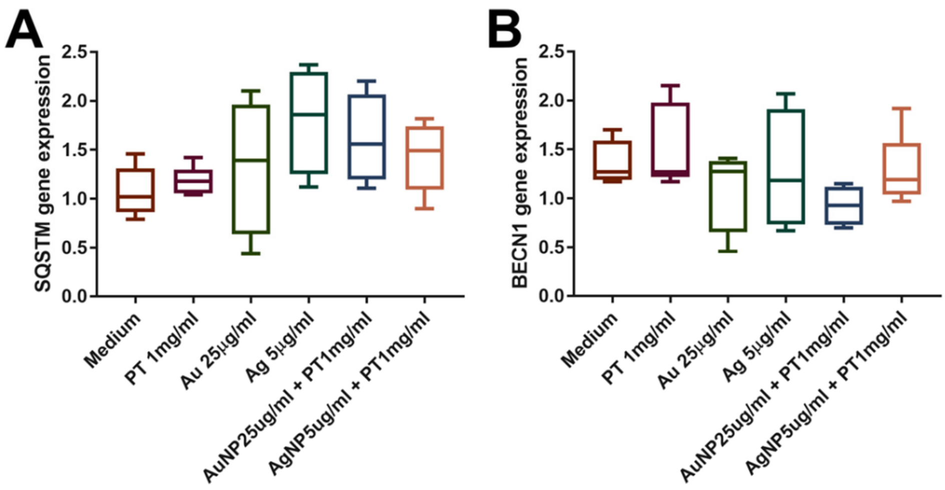

Figure S2. *mRNA expression of SQSTM (A) and BECN1 (B) genes. Box plots represent median, 25th, and 75th percentiles. Whiskers indicate 5th and 95th percentiles.*

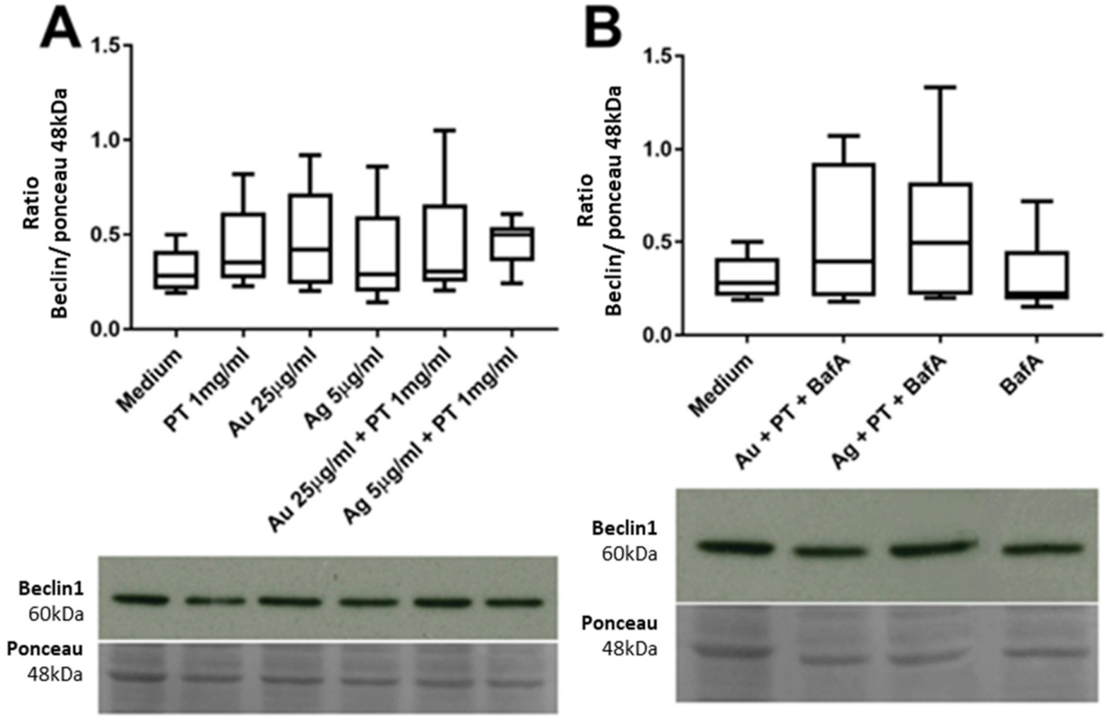

Figure S3. *Quantification of Beclin-1 protein level with western blot assays, after the stimulation with PT and mNPS (A) or the combination of PT, mNPs and BafA (B). The target protein was normalized on Ponceau staining in correspondance of the 48kda band. Box plots represent median, 25th, and 75th percentiles. Whiskers indicate 5th and 95th percentiles. Western blot image representative of at least three independent experiments.*

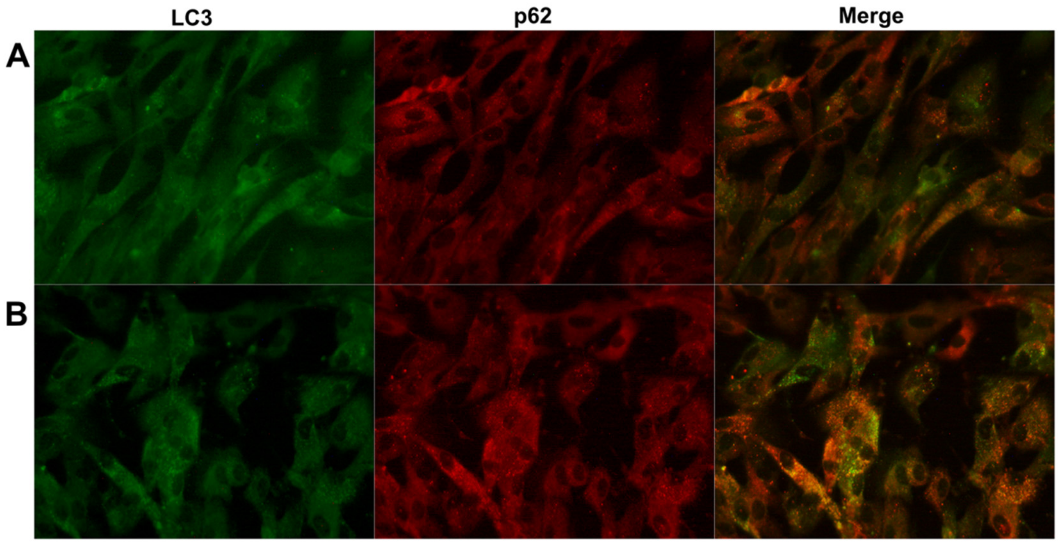

Figure S4. *Indirect immunofluorescence of HIEC cells after 24 hours treatment with AuNPs 25µg/ml (A) or AgNPs 5µg/ml (B). Green signal shows LC3, Red signal p62, yellow signal shows the merge between the two. (40x)*
